# Post-Treatment Neck Dissection of Tonsillar and Base of Tongue Squamous Cell Carcinoma in the Era of PET-CT, HPV, and p16

**DOI:** 10.3390/v14081693

**Published:** 2022-07-30

**Authors:** David Landin, Anders Näsman, Sara Jonmarker Jara, Lalle Hammarstedt-Nordenvall, Eva Munck-Wikland, Tina Dalianis, Linda Marklund

**Affiliations:** 1 Department of Clinical Science, Intervention and Technology, Department of Oto-Rhinolaryngology, Head and Neck Surgery, Karolinska University Hospital, Karolinska Institute, 17164 Stockholm, Sweden; david.landin@ki.se (D.L.); lalle.hammarstedt@ki.se (L.H.-N.); eva.munck-wikland@ki.se (E.M.-W.); 2 Medical Unit Head Neck, Lung and Skin Cancer, Karolinska University Hospital, 17164 Stockholm, Sweden; 3 Department of Oncology, Pathology, Karolinska Institute, 17164 Stockholm, Sweden; anders.nasman@ki.se; 4 Department of Clinical Pathology, Karolinska University Hospital, 17164 Stockholm, Sweden; 5 Department of Neuroradiology, Karolinska University Hospital, 17164 Stockholm, Sweden; sara.jonmarker-jaraj@regionstockholm.se; 6 Department of Surgical Sciences, Section of Otolaryngology and Head and Neck Surgery, Uppsala University, 75105 Uppsala, Sweden

**Keywords:** tonsillar squamous cell carcinoma, base of tongue squamous cell carcinoma, oropharyngeal squamous cell carcinoma, human papillomavirus, PET-CT, metastasis

## Abstract

Human-papillomavirus (HPV)-positive tonsillar and base of tongue carcinomas (TSCC/BOTSCC) are rising in incidence and treatments with radiotherapy, chemoradiotherapy (RT/CRT), and neck dissections (NDs) have several side effects. Therefore, an improved selection of patients needing salvage NDs would be beneficial. We examined the prevalence and localisations of viable tumour cells in neck lymph nodes in patients post-RT/CRT, identified by fluorodeoxyglucose positron-emission tomography with computer-tomography (FDG PET-CT), with a focus on HPV-associated tumours. Patients with 217 TSCC/BOTSCC with tumours assessed for HPV-DNA and p16^INK4a^ undergoing FDG PET-CT 12 weeks after treatment and/or an ND were included. The FDG PET-CT data were compared with the findings in the pathology report after the ND. In total, 36/217 (17%) patients were selected for an ND due to positive findings in post-treatment FDG PET-CT. Of these, 35/36 were HPV-associated, 10/36 (28%) had viable tumour cells in the pathology reports of the neck specimen, and 8/10 (80%) were consistent with the FDG PET-CT findings, while 2/36 (5%) were missed by FDG PET-CT. We conclude that FDG PET-CT 12 weeks after RT/CRT is useful, but not completely reliable for finding all the metastases of HPV-associated TSCC/BOTSCC. Nonetheless, our data indicate that an ND could be more selectively guided by FDG PET-CT.

## 1. Introduction

In recent years, there has been a dramatic increase in oropharyngeal squamous cell carcinomas (OPSCC) in many Western countries, mostly due to an increase in human-papillomavirus (HPV)-induced cases [1,2,3,4,5]. The vast majority of OPSCC cases are tonsillar and base of tongue squamous cell carcinomas (TSCC and BOTSCC, respectively), and patients with such tumours have a better prognosis than patients with corresponding HPV-negative cancers [6,7]. This has led to the introduction of a new staging protocol for OPSCC in the latest American Joint Committee on Cancer/Union for International Cancer Control (AJCC-8/UICC-8). In the new protocol, p16 ^INK4a^ overexpression in >70% of the cells (p16+) is used as a surrogate marker for the presence of HPV, and patients with OPSCC are staged differently depending on p16 status [8]. Furthermore, due to the favourable prognosis of patients with HPV-mediated OPSCC, there have been efforts to de-escalate today’s more intensified treatments in order to reduce therapy-related side effects and complications [9]. However, these efforts have not always been successful, and in a systematic review and meta-analysis, it was shown that there was a concrete risk in offering suboptimal treatment to patients [9].

For a long time, radiotherapy (RT) and chemoradiotherapy (CRT) have been the primary treatments of OPSCC at many medical centres, especially in Sweden [6]. CRT is an effective treatment but comes with considerable side effects [10]. Furthermore, OPSCC, especially if defined as HPV-positive, often presents with nodal disease in the neck (N+), and different strategies have been used for the post-CRT management of such diseases [11,12].

Recently, positron-emission tomography with computer tomography (PET-CT) has gained more attention as an option for post-treatment surveillance in head and neck cancers. In 2016, a randomized controlled study was published comparing fluorodeoxyglucose (FDG) PET-CT with computer tomography (CT) and neck dissection [13]. Survival in the two groups was similar. However, since FDG PET-CT is more cost-effective, many medical centres, including ours, have introduced a similar post-treatment FDG PET-CT protocol 12 weeks after finishing RT/CRT [13,14]. Although a negative FDG PET-CT scan is highly reliable for showing the absence of residual cervical node disease, the incidence of a false-positive FDG PET-CT finding is still relatively high, even though the investigation takes place 12 weeks after the completed RT/CRT [15,16]. There are also indications that FDG PET-CT has a lower positive predictive value in HPV-related OPSCC [17]. Additional studies are therefore warranted.

Since 2018, OPSCC patients at the Karolinska University Hospital without complete response in the neck, i.e., defined by size and/or intensity of an FDG-uptake in the lymph nodes upon the FDG PET-CT recording and protocol 12 weeks after completed RT/CRT, have been scheduled for a selective neck dissection (ND). Post-treatment NDs in these cases often include the neck regions 1–4/5. The impact of RT/CRT on the tissue of the neck makes NDs post-RT/CRT far more surgically complicated than NDs done on an untreated neck and escalates the risk for long-term complications, such as tightness of the neck [10]. A more limited ND, including only lymph nodes without complete response according to the FDG PET-CT protocol, would reduce the risk of surgical complications as well as reduce sequelae in the neck region affected by both surgery and RT/CRT. 

The focus of this study was therefore to examine the prevalence of viable tumour cells in metastases at the post-RT/CRT treatment of HPV-positive TSCC/BOTSCC in patients who had an ND due to a FDG PET-CT result (showing an increased FDG-uptake or enlarged lymph nodes) 12 weeks after completing treatment. In addition, the localisation of remaining viable tumour cells in the histopathological report of the surgical neck specimen in relation to the FDG PET-CT result was assessed. Finally, we examined the prevalence of viable cancer cells located in lymph nodes other than those with an increased FDG uptake or in enlarged lymph nodes presented in the FDG PET-CT scan. Acquiring such knowledge could be helpful in revealing if a reduced ND guided by FDG PET-CT is a safe post-treatment alternative for patients, especially those with HPV-associated TSCC/BOTSCC.

## 2. Materials and Methods

### 2.1. Patients and Tumour Samples 

Through the Swedish Cancer Registry, between 2017 and 2021, in the counties of Stockholm/Gotland, Sweden, 439 patients were identified as diagnosed with TSCC and BOTSCC with regional metastases (N+) (TSCC: ICD-10 C09.0, C09.8, and C09.9; BOTSCC: C01.9). During the above time period, patients treated with intention to cure in the Stockholm region were primarily treated with RT consisting of 68 Gy given for 6 weeks, or CRT consisting of cisplatin and RT as presented above, and in some cases with cetuximab. Only patients with T1 without N or M were treated with surgery alone, and this was not the case for any of the 39 patients listed further below.

In this study, patients without post-treatment PET-CT or salvage NDs were excluded (n = 222). Consequently, 217 patients with TSCC and BOTSCC treated with intention to cure and who underwent post-treatment FDG PET-CT or salvage NDs were included in the initial analysis (Table 1). The patient charts of these 217 patients were assessed for age, sex, and tumours assessed for the presence of HPV-DNA and p16^INK4a^ overexpression (defined as p16+ when >70% were stained positive). The latter two were analysed as described before [4,6]. FDG PET-CT results with an intense FDG uptake at 12 weeks after CRT and/or enlarged lymph nodes in the neck were classified as incomplete nodal responses. Mild or no FDG uptakes in enlarged nodes or a mild FDG uptake in normal-sized nodes were considered to be an equivocal response [18]. All other FDG PET-CT results were considered to show a complete response. In our cohort, 39 patients with an incomplete or equivocal response in lymph nodes in the neck (where 36 had FDG PET-CT performed and three due to enlarged nodes) and a complete response in the primary site underwent NDs (Table 2). The selection for NDs after oncological treatment for these patients was thus performed according to the current guidelines [18].

The 39 patients who underwent salvage NDs were further assessed for TNM 7 and 8, smoking, WHO performance status, the result of the FDG PET-CT scan, and treatment classified as RT or CRT. The smoking data were obtained from the charts and characterised as current smoker, never smoker, or former smoker. Consequently, NDs were performed on 39 patients, of which 36 underwent prior FDG PET-CT, and the results of post-treatment FDG PET-CT were compared with the pathology reports from the neck specimen regarding the location of metastases and the presence of viable tumour cells. The study was conducted in accordance with ethical permissions 2009/1278-31/4 and 2017/1035-31/2 from the Stockholm Regional Ethical Review Board. 

### 2.2. Statistics

The correlation between the FDG PET-CT findings and the presence of histopathological viable cancer was analysed using SPSS Statistics (SPSS Statistics for Mac, Version 28, IBM Corp, Armonk, NY, USA), estimating the 95% confidence interval. HPV-positive and -negative tumours were calculated in the surgery and non-surgery groups. Fisher’s exact test was used to calculate possible significant differences at the *p* < 0.05 level between TSCC and BOTSCC in Table 1, Table 2 and Table 3.

## 3. Results

### 3.1. Patients and Their Characteristics

In total, 217 patients with TSCC/BOTSCC who underwent post-treatment FDG PET-CT and/or NDs between 2017 and 2021 and their tumours were evaluated, as described above in more detail (Table 1). Of the tumours, 202/217 (93%) were HPV-DNA-positive according to PCR testing and the vast majority were subtyped as HPV16 (n = 169), while the second most common subtype was HPV33 (n = 20). Twelve samples were HPV-DNA-negative, and three samples were not tested. One hundred and eighty-seven (86%) tumour samples were p16+, 12 (6%) were p16-negative (p16−), and 18 (8%) did not have an available test result (Table 1). 

There were no statistical differences (Fisher’s exact test) between TSCC and BOTSCC regarding the parameters listed in Table 1.

### 3.2. Patients and Their Treatments

In total, 39/217 (18%) patients were treated with NDs. Of these, 36/217 (17%) NDs were due to the results of FDG PET-CT, and 3/217 (1%) were due to enlarged lymph nodes. All these patients are depicted in Table 2, and there were no significant differences (Fisher’s exact test) between TSCC and BOTSCC regarding the listed parameters.

The most common neck regions included in the NDs were the regions 1–4 (n = 15, 38%), followed by the regions 1–5 (n = 13, 33%), regions 2–4 (n = 4, 10%), and other types of regions (n = 7, 18%). Thirty-seven (95%) of the patients who underwent NDs had HPV-DNA-positive and p16+ cancers, while of the remaining two patients, one had an HPV-DNA-positive and p16− cancer, and the other an HPV-DNA-negative and p16− cancer. 

More specifically, of the 36/39 (92%) patients selected for surgery due to FDG PET-CT criteria, 35 had HPV-DNA-positive and p16+ tumours, while the remaining patient had an HPV-DNA-positive, but p16− tumour. Among those patients, 30/36 (83%) tumours demonstrated an increased FDG uptake, while 6/36 (17%) tumours were selected for an ND based on an enlarged nodal size without an elevated FDG uptake.

The additional three who received post-treatment RT/CRT NDs did not have a prior FDG PET-CT scan but had enlarged lymph nodes with regular CT or at a clinical examination 12 weeks after treatment. Of these three, two had HPV-DNA-positive and p16+ tumours, while the remaining one had an HPV-DNA-negative and p16− tumour. These three patients were later shown to have viable tumour cells upon histopathological examination and were subsequently excluded from further analysis and discussion when comparing FDG PET-CT results and the histomorphological assessment of the neck specimen.

### 3.3. Patients Examined with FDG PET-CT and Undergoing ND

As mentioned above, in total, 36 patients were selected for NDs based on the FDG PET-CT results; for further details, see Table 3.

There were no significant differences (Fisher’s exact test) between TSCC and BOTSCC regarding the listed parameters in Table 3. Of the above patients, 26/36 (72%) had no signs of viable tumour cells in the histopathological examination of the neck specimen. In this regard, there was no statistical difference observed between TSCC and BOTSCC: 15/20 (75%) and 11/16 (69%), respectively, as already mentioned. 

Of the 10/36 (28%) patients with viable tumour cells in the histopathological examination, 8/36 (22%) were consistent with the FDG PET-CT results with signs of active metastases at the corresponding localisations. In the remaining 2/36 (6%) patients with viable tumours (both HPV-DNA-positive and p16+), the tumour cells were found in cervical lymph nodes other than those indicated by the FDG PET-CT results. More specifically, one patient had T4N2M0 TSSC. The viable tumour cells were seen at the same localisation as the metastases/nodes seen in the pre-treatment CT but did not show an FDG uptake and was at the time too small to meet the post-treatment PET-CT criteria for an ND. The viable tumour cells found in the ND of the other patient, T2N1M0 TSCC, were located in a contralateral neck region without a known metastasis before treatment and had thereby not received therapeutic radiation. To summarise, in total, two sites with viable cancer were missed with FDG PET-CT, resulting in a sensitivity of 0.80 (95% CI: 0.44–0.96). However, due to the limited number of patients in this study and the variation in CI, this sensitivity should not be overinterpreted.

The majority, 7/10 (70%) of patients with viable tumour cells in the NDs, showed a high FDG uptake, while 3/10 (30%) did not have a markedly increased FDG uptake and were selected for an ND due to enlarged size criteria. Nonetheless, the proportion of viable tumour cells in the neck specimen was comparable between patients selected for an ND due to an increased FDG uptake or an enlarged size: 8/30 (26%) and 2/6 (33%), respectively. 

## 4. Discussion

In this study, 36/217 (17%) patients with N+ TSCC/BOTSCC underwent post-RT/CRT treatment NDs due to the results of FDG PET-CT. Virtually all patients were HPV-associated (all were HPV-DNA-positive and 35/36 p16+). Of these, 26/36 (72%) had no signs of viable tumour cells, while 10/36 (28%) showed viable tumour cells in the pathology report of the neck specimen, and the majority of the latter 8/10 (80%) were consistent with the FDG PET-CT examination. Consequently, two sites with viable cancer were missed with FDG PET-CT. In one patient with inconsistent data, the viable metastasis was in the same location as a known metastasis in pre-treatment CT but did not show an FDG uptake and was too small to meet the post-treatment FDG PET-CT criteria for an ND. In the other, the metastasis found in the ND was located in a contralateral neck region without a known metastasis before treatment. Thus, only one patient presented viable cancer in a node undetected with FDG PET-CT, 1/35 (2.8%) in the ipsilateral neck side, and was treated with an ND after RT/CRT. 

These findings indicated that reducing the regions included for post-RT or a CRT-ND should be done with caution, but the data also implicate that perhaps not all ipsilateral neck regions must be included. A reduced ND, only including the lymph nodes indicating the remaining cancer through FDG PET-CT, or alternatively, the neck regions containing metastases in pre-treatment CT, may be considered. 

TSCC and BOTSCC are frequently caused by HPV, and in the present study, 93% of the cases were HPV-DNA-positive and 86% p16+. With an increase in vaccination and decrease in smoking, there may be a decrease in the incidence of TSCC and BOTSCC in the future [19]. However, this may take a few decades, and the need for optimized treatment for this group of patients remains. 

As mentioned above, in this cohort, 36/217 (17%) N+ TSCC/BOTSCC patients underwent an ND after surveillance with FDG PET-CT 12 weeks after completing RT/CRT. Comparing this with the surgery for all patients, which previously was the treatment of choice at many cancer centres, we suggest that FDG PET-CT has clear benefits and may spare many patients an unnecessary ND [20]. Nevertheless, only 10/36 (28%) of all patients with an increased FDG uptake or enlarged lymph nodes, according to the present criteria for post-treatment NDs, showed viable cancer in the histopathologic examination in our study. Our data were therefore in line with previous studies that have shown slightly lower positive predictive values for post-treatment PET-CT 12 weeks after treatment for p16+ OPSCC compared with other head and neck cancers [21,22,23,24,25]. 

However, there were limitations in our study. First, the number of study subjects was limited, which poses a risk for an overinterpretation of the data. Furthermore, although our focus was to evaluate post-treatment FDG PET-CT in patients with HPV-DNA-positive and p16+ TSCC/BOTSCC, it may have been better to examine whether similar or different findings would have been obtained in HPV-non-associated TSCC/BOTSCC. However, this issue could not be explored, because the vast majority of the tumours were HPV-associated. Moreover, it may have been beneficial in case of uncertainty to compare the obtained FDG PET-CT data with fine needle aspiration cytology or a control ultrasound after another 6 weeks to identify progress. The latter is cost-efficient and may have spared some patients an unnecessary ND. 

To manage the relatively low predictive value of post-treatment FDG PET-CT, some previous studies have suggested that when there are signs of metastases according to PET-CT after 12 weeks, a repeated FDG PET-CT scan should be performed to improve accuracy [23]. Alternatively, a prolonged interval between RT/CRT and the first post-treatment PET-CT could be considered for HPV-DNA p16+ OPSCC [23,24,25]. Our study was in line with these previous studies, and we agree that extending the time frame for FDG PET-CT to >12 weeks after treatment and performing a repeated FDG PET-CT scan afterward may be useful [23,24,25]. This would be particularly relevant for patients with HPV-positive tumours, where the prognosis in general is very good. 

In the future, an alternative to post-treatment assessment for patients with HPV-DNA-positive cancer may be measuring circulating tumour HPV-DNA (ctHPVDNA) [26,27,28]. Possibly combining FDG PET-CT and ctHPVDNA may be even more useful. Furthermore, future studies may compare the sensitivity of using post-treatment FDG PET-CT at 12 weeks and 6 months [23] with clinical palpation of the neck by an experienced examiner. The comparability of the thorough clinical examination with new, more advanced methods is a question of significance in areas with limited access to PET-CT and advanced laboratory techniques. 

Larger studies are warranted to further evaluate the value of post-treatment FDG PET-CT for TSCC/BOTSCC and whether there are differences between HPV-positive and -negative cancers, as well as defining the optimal timing. 

## 5. Conclusions

We concluded that post-treatment FDG PET-CT is useful, but not completely reliable in finding all metastases in patients with HPV-DNA-positive p16+ TSCC/BOTSCC. Most, i.e., 8/10 patients, but not all patients (2/10) with HPV-associated cancer had viable cancer in lymph nodes identified with FDG PET-CT in the neck specimen. Consequently, 2/36 of the patients who underwent a post-treatment ND had viable tumour cells in lymph nodes other than those identified with post-FDG PET. The ones missed were enlarged in pre-treatment CT or initially wrongly staged. Nevertheless, the data implicate that a post-treatment ND should be more selective, either focusing on FDG-positive lymph node regions or neck regions containing metastases in pre-treatment CT. This can putatively reduce the risk of surgery, allow for a decreased surgical time, and minimize the area of the neck affected by the long-term side effects of treatment. Still, further studies including a prolonged period between treatment and FDG PET-CT or combining FDG PET-CT with ctHPVDNA are warranted.

## Figures and Tables

**Table 1:** Patient and tumour characteristics.

		TSCC n (%)	BOTSCC n (%)	Total n (%)
Number of patients		139 (64%)	78 (36%)	217
Median age	Years	62	63	63
Sex	Female	40 (29%)	17 (22%)	57 (36%)
	Male	99 (71%)	61 (78%)	160 (64%)
p16 overexpression	No	3 (2%)	9 (12%)	12 (6%)
	Yes	121 (87%)	66 (84%)	187 (86%)
	Not known	15 (11%)	3 (4%)	18 (8%)
HPV DNA status	Negative	4 (3%)	8 (10%)	12 (6%)
	HPV 16	115 (83%)	54 (69%)	169 (78%)
	HPV 33	12 (9%)	8 (10%)	20 (9%)
	HPV 35	3 (2 %)	4 (5%)	7 (3%)
	HPV 18	1 (1%)	1 (1%)	2 (1%)
	HPV 58	2 (1%)	0 (0%)	2 (1%)
	Mixed	1 (1%)	1 (1%)	2 (1%)
	Not known	1 (1%)	2 (3%)	3 (1%)

**Table 2:** Patients treated with a neck dissection (ND) and their characteristics.

		TSCC ^1^ n (%)	BOTSCC ^2^ n (%)	Total n (%)
Number of patients		22 (56%)	17 (44%)	39 (100%)
Median age	Years	61	64	63
Sex	Female	5 (23%)	4 (24%)	9 (23%)
	Male	17 (77%)	13 (76%)	30 (77%)
p16 overexpression	No	1 (5%)	2 (12%)	3 (8%)
	Yes	19 (86%)	13 (76%)	32 (82%)
	Not known	2 (9%)	2 (6%)	4 (10%)
HPV DNA status	Negative	1 (5%)	1 (6%)	2 (5%)
	HPV 16	15 (68%)	13 (69%)	28 (72%)
	HPV 33	3 (14%)	2 (10%)	5 (13%)
	HPV 35	1 (5%)	1 (10%)	2 (5%)
	HPV 18	1 (5%)	0 (0%)	1 (3%)
	HPV 58	1 (5%)	0 (0%)	1 (3%)
T (AJCC 7th Edition)	T1	8 (36%)	8 (47%)	16 (41%)
	T2	7 (32%)	7 (41%)	14 (36%)
	T3	2 (9%)	0 (0%)	2 (5%)
	T4a	5 (23%)	2 (12%)	7 (18%)
	T4b	0 (0%)	0 (0%)	0 (0%)
N (AJCC 7th Edition)	N0	0 (0%)	0 (0%)	0 (0%)
	N1	2 (9%)	0 (0%)	2 (5%)
	N2a	0 (0%)	1 (6%)	1 (3%)
	N2b	15 (68%)	12 (71%)	27 (69%)
	N2c	3 (14%)	3 (18%)	6 (15%)
	N3	2 (9%)	1 (6%)	3 (8%)
	NX	0 (0%)	0 (0%)	0 (0%)
M (AJCC 7–8th Edition)	M0	21 (95%)	16 (94%)	37(95%)
	M1	0 (0%)	0 (0%)	0 (0%)
	MX	1 (5%)	1 (6%)	2 (5%)
T (AJCC 8th Edition)	T1	8 (36%)	8 (47%)	16 (41%)
	T2	7 (32%)	7 (41%)	14 (36%)
	T3	2 (9%)	0 (0%)	2 (5%)
	T4	4 (18%)	2 (12%)	6 (15%)
	T4a	1 (5%)	0 (0%)	1 (3%)
	T4b	0 (0%)	0 (0%)	0 (0%)
N (AJCC 8th Edition)	N0	0 (0%)	0 (0%)	0 (0%)
	N1	15 (68%)	9 (53%)	24 (62%)
	N2	3 (14%)	4 (24%)	7 (18%)
	N2a	0 (0%)	0 (0%)	0 (0%)
	N2b	1 (5%)	1 (6%)	2 (5%)
	N2c	0 (0%)	0 (0%)	0 (0%)
	N3	3 (14%)	3 (18%)	6 (15%)
	NX	0 (0%)	0 (0%)	0 (0%)
Smoking	Current	3 (14%)	3 (18%)	6 (15%)
	Never	10 (45%)	6 (35%)	16 (41%)
	Former	9 (41%)	8 (47%)	17 (44%)
Performance status (WHO/ECOG)	0	19 (86%	15 (88%)	34 (87%)
	1	2 (9%)	1 (6%)	3 (8%)
	2	1 (5%)	1 (6%)	2 (5%)
	3	0 (0%)	0 (0%)	0 (0%)
Treatment	RT	9 (41%)	5 (29%)	14 (36%)
	CRT	13 (59%)	12 (71%)	25 (64%)
Regions in neck dissection	1 to 5	9 (41%)	4 (24%)	13 (33%)
	1 to 4	8 (36%)	7 (41%)	15 (38%)
	2 to 4	3 (14%)	1 (6%)	4 (10%)
	other	2 (9%)	5 (29%)	7 (18%)

^1^ TSCC: tonsillar squamous cell carcinoma, ^2^ BOTSCC: base of tongue squamous cell carcinoma.

**Table 3:** Patients with data on NDs performed after a positive FDG PET-CT scan.

Patients with NDs and PET-CT		TSCC	BOTSCC	Total
Number of patients		20 (56%)	16 (44%)	36 (100%)
Result of histopathology in relation to a positive PET-CT result	No cancer	15 (75%)	11 (69%)	26 (72%)
	Viable cancer in the same region as the FDG PET-CT result	3 (15%)	5 (31%)	8 (22%)
	Viable cancer in a different region than the FDG PET-CT result	2 (10%)	0 (0%)	2 (6%)

## Data Availability

Not applicable.
